# The SIRAH-CoV-2 Initiative: A Coarse-Grained Simulations' Dataset of the SARS-CoV-2 Proteome

**DOI:** 10.3389/fmedt.2021.644039

**Published:** 2021-02-15

**Authors:** Pablo G. Garay, Exequiel E. Barrera, Florencia Klein, Matias R. Machado, Martín Soñora, Sergio Pantano

**Affiliations:** Biomolecular Simulations Group, Institut Pasteur de Montevideo, Montevideo, Uruguay

**Keywords:** coarse-grain, simulation, data sharing, molecular dynamics, virus, COVID-19, microsecond

## Introduction

During the last decades, the broad community of computational biophysicist/biochemists has developed computational tools to quickly test molecular hypotheses, support, complement, and even substitute experimental data in a reliable and reproducible fashion. As a by-product of these advances, enormous amounts of data are being generated (1). Unfortunately, good practices about data archiving, documenting, and sharing are not in pace with the formidable capacity to produce information. This often results in suboptimal utilization of efforts and resources, leaving authentic “data treasures” undiscovered. This redounds in a useless replication of work, which often times is only needed as input for further investigation rather than representing an objective themselves (1). It, therefore, becomes increasingly important to make computational biophysics data publicly available, searchable, and downloadable, adhering to the “FAIR” principles (2).

Among many others, the European community has advanced a large and coordinated initiative, the European Open Science Cloud (EOSC), which is aimed at sharing and re-using scientific content increasing transparency and accountability. OpenAIRE is a socio-technical infrastructure for scholarly communication and Open Science (3). It offers data store ensuring long-term preservation of relatively “big size” datasets. Among others, the Zenodo database (4) provides a simple and fast upload system, with the possibility to immediately obtain a DOI identifier for each data set, including the option to update data sets separately.

Early in 2020, the COVID-19 pandemic pervaded virtually all personal and scientific activities with extensive lockdown regimes in most countries across the world. In response to this extraordinary context, the entire scientific community devoted massive efforts to study SARS-CoV-2 at basic and applied levels. The Biocomputing community was not an exception and showed a strong commitment endorsed by hundreds of groups around the globe (5). Many researchers reoriented their priorities, offering a swift response to the emergency, providing fresh structural and dynamical perspectives on viral variability, drug targets, effect of mutations, etc. (5). As a result, only a few months after the beginning of the pandemic, it was possible to find many data-sharing initiatives scattered in different portals and repositories.

**Figure 1:**
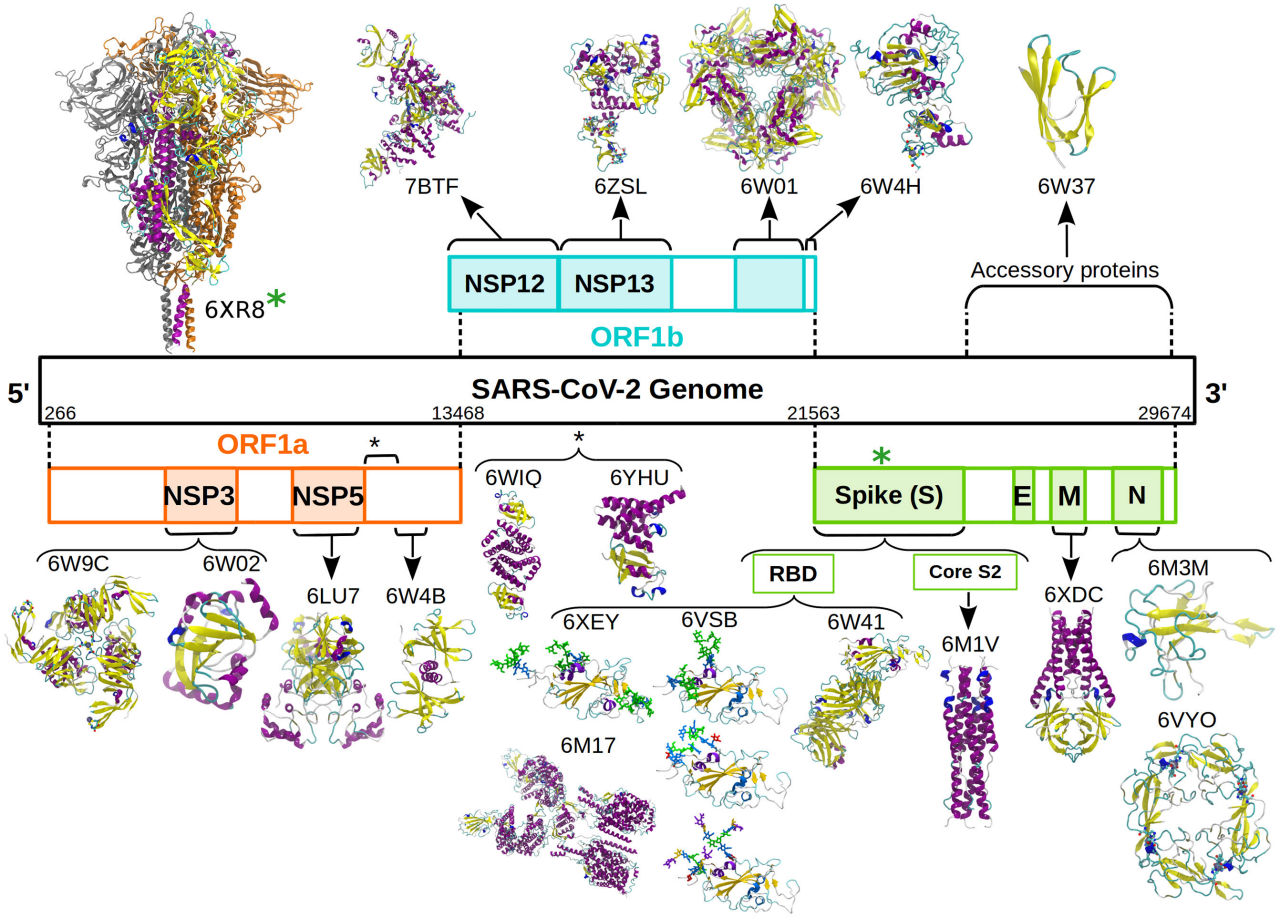
Schematic representation of the SARS-CoV-2 genome and associated proteins. All the proteins simulated are presented as cartoon and colored according to their secondary structure following the standard VMD color code (6). Glycans are presented as sticks and colored according to the SNFG color scheme (7). The D614G mutation was introduced in the soluble domain of the wild type Spike protein (green asterisk).

In this context, our group undertook the initiative of simulating and sharing the raw data of coarse-grained (CG) simulations of the SARS-CoV-2 proteins reported in the PDB, in the apo state. Figure 1 shows the representative structures reported in the PDB database until October 30, 2020. We named this “*The SIRAH-CoV-2 Initiative*,” which was carried out in collaboration with the Uruguayan National Center for Supercomputing, ClusterUY (https://www.cluster.uy) (8). The raw data for individual CG Molecular Dynamics (MD) simulations is available from the Zenodo database (9–28).

## Methods

Simulations were performed using the SIRAH force field 2.0 (29) running with the Amber18 suite (http://ambermd.org) at ClusterUY. Interaction parameters for bound divalent cations and glycans were reported by Klein et al. (30) and Garay et al. (31), respectively.

Coordinates were downloaded from the PDB database (PDBs id: 6VYO, 6W01, 6LU7, 6W02, 6W4B, 6M3M, 6W9C, 6W4H, 6W41, 6YHU, 6W37, 6WIQ, 7BTF, 6M17, 6VSB, 6M1V, 6XDC, 6ZSL, 6XEY, 6XR8). Non-protein, non-glycan molecules and ions not coordinated by proteins were removed (e.g., water and molecules present in crystallization buffers). When deemed necessary, missing residues were reconstructed with ModLoop (32). The D614G mutation in the SARS-CoV-2 Spike protein was introduced on the wild type structure (PDB id: 6XR8) by simply deleting the side chain of Asp614 and renaming the residue. Only in this particular case, missing loops were completed using SWISS-MODEL at https://swissmodel.expasy.org. All structures were protonated using PDB2PQR (33) at a pH = 7. The orientation of the protein in PDB id 6XDC was set according to the OPM database (https://opm.phar.umich.edu/proteins/5172), with a pre-equilibrated patch containing POPC, POPE, and POPS phospholipids in a 1:2:1 relation according to the experimental data (34). Interaction parameters for lipids were taken from Barrera et al. (35). The glycosylation trees were added/completed (in PDB ids 6VSB and 6XEY) using the Glycan Modeler & Reader utility from CHARMM-GUI (36).

All parameters are available for download from the SIRAH force field web page (http://www.sirahff.com).

Protonated structures were mapped to CG with SIRAH Tools (37). Solutes were centered in an octahedral box filled with pre-equilibrated SIRAH's CG water molecules named WT4 (38). An ionic strength of 0.15 M was set by randomly replacing WT4 molecules with Na^+^ and Cl^−^ CG ions (39).

Since SIRAH uses a Hamiltonian common to any atomistic MD simulation, the 6–12 terms used to treat Lennard-Jones interactions might lead to large repulsions if initial structures suffer from clashes. Because of this, gentle initialization protocols aimed to resolve steric clashes are strongly recommended.

The simulation protocol consisted of:

1. Solvent and side chains relaxation by 5,000 steps of energy minimization, imposing positional restraints of 2.4 kcal mol^−1^ Å^−2^ on backbone beads corresponding to the nitrogen and carbonylic oxygen (named GN and GO, respectively). When Zinc or glycans are present, these restrains also apply to the beads corresponding to the metal ions and sugar rings (named ZnX, GO2, GNac, GO3, GO4, GC6, GC1, and GO7).
2. Full system relaxation by 5,000 steps of unrestrained energy minimization.
3. Solvent equilibration by 5 ns of MD in the NVT ensemble at 300 K, imposing positional restraints of 2.4 kcal mol^−1^ Å^−2^ on the whole protein and glycans and Zinc ions, when present.
4. Biomolecule relaxation by 25 ns of MD in the NVT ensemble at 300 K, imposing positional restraints of 0.24 kcal mol^−1^ Å^−2^ on the mentioned beads.
5. Same as step 4 with the position restrains of 0.12 kcal mol^−1^ Å^−2^.
6. Production simulation in the NPT ensemble at 300 K and 1 bar.

We used a time step of 20 fs and a direct cutoff of 1.2 nm for non-bonded interactions and Particle Mesh Ewald (PME) for long-range electrostatics (40, 41). Snapshots were recorded every 200 ps. PME was calculated at every integration step owing to code restrictions, and the neighbor list was updated whenever any atom had moved more than one-half a non-bonded “skin” of 0.2 nm. A Fourier spacing close to 0.1 nm was used. The whole system was coupled to a Langevin thermostat (42) with a collision frequency of 50 ps^−1^ and to a Berendsen barostat (43) with a relaxation time of 1 ps.

The multimicroseconds CG MD trajectories of SARS-CoV-2 proteins include the information required to visualize, analyze, and backmap on VMD (6). Each entry is constituted by three subsets of data associated with the same CG MD simulation. The first set (referred as raw data) contains the system's topology, starting configuration, simulation report, last checkpoint, and trajectory in AMBER format and allows continuing the simulation. The second subset contains a “stripped” version of the MD, not including solvent, while the third contains a “skipped” trajectory with one frame every 10 ns.

Since CG beads in SIRAH are mapped from atoms' position, it is possible to get direct measures from the trajectories using VMD tools. These include Root Mean Square Deviation (RMSD), radius of gyration, etc. Moreover, a tcl script corresponding to SIRAH Tools is present in each tar file of the dataset that performs additional analyses and secondary structure content from the VMD's Tcl/Tk console (6). Typing sirah_help displays all available options. It enables macros for visualizing and coloring residue types, the element corresponding to each CG bead, among others. This tool is totally compatible with all the functions on VMD and used the same color schemes. Provided that Amber Tools (44) is locally installed, it is possible to obtain pseudoatomistic structural models at every point of the trajectory.

## Utilization of the Data

We started this initiative to provide our colleagues with a complete and homogeneous set of CG MD simulations that could facilitate the analysis of large-scale dynamics of SARS-CoV-2 proteins.

**Figure 2:**
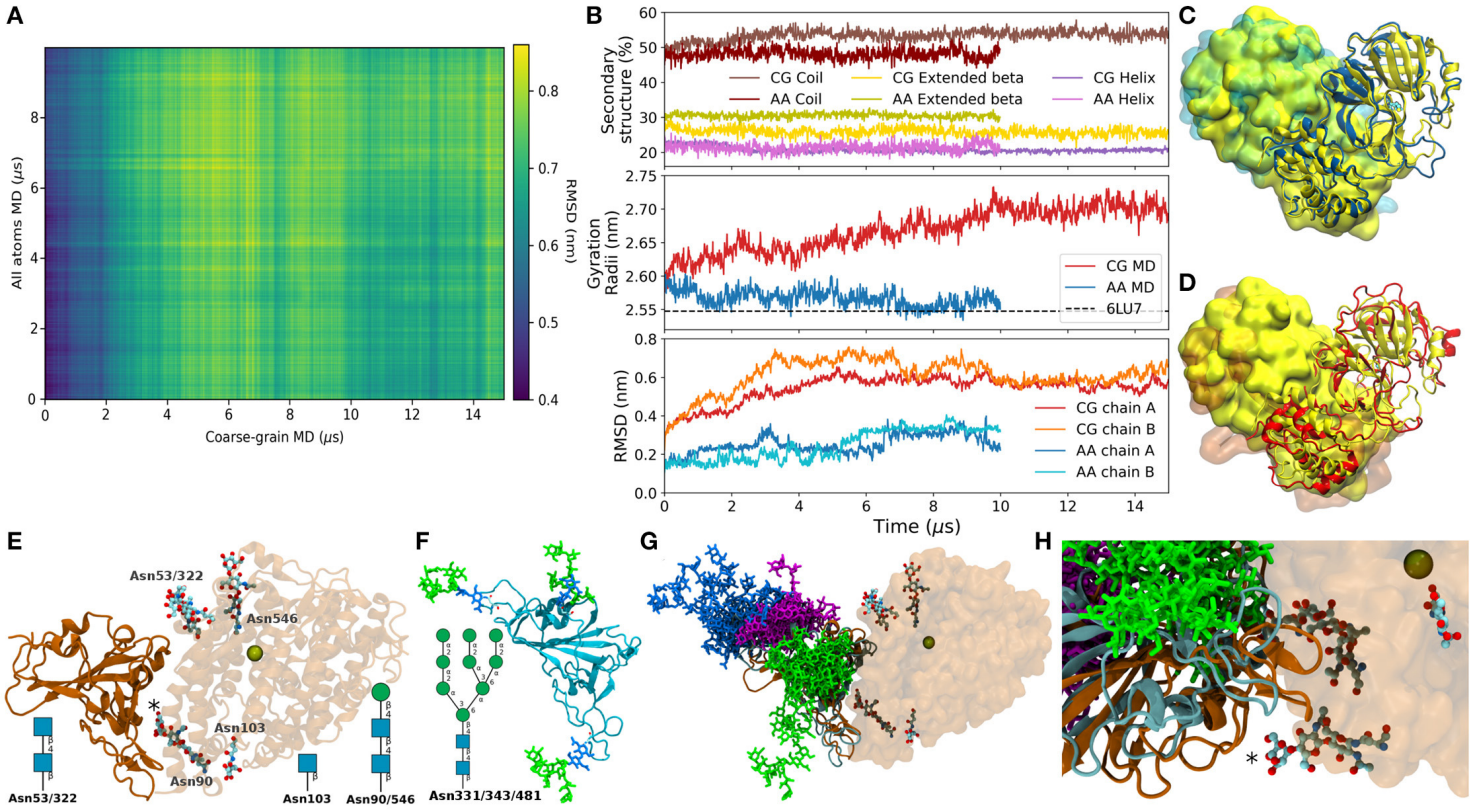
**(A)** Bi-dimensional RMSD of the Cα between the atomic trajectory and the backmapped conformations from the CG trajectory. **(B)** Top: Percentage of secondary structure content. Middle: Gyration Radii of the whole proteins from the atomic (blue), and backmapped trajectory in red. Bottom: RMSD of the Cα between the trajectories and the experimental structure, separated by chains. **(C)** Superposition between the experimental structure (yellow) and the last conformation of the atomic trajectory (blue). Chains A are presented as cartoon and chains B as presented as surface. **(D)** Same as **(C)** with the backmapped structure of the last frame of the SIRAH trajectory in red. **(E)** X-ray structure PDB 6VW1 in cartoon representation, ACE2 is semitransparent, glycosylations are show as sticks colored according to element. The Zinc ion present in the binding site is shown as a space-filling sphere. The asterisk marks the glycosylation at Asn90 on ACE2. The N-glycosylation solved by X-ray on each position are indicated schematically. **(F)** Starting conformer of the RBD glycosylated at Asn331, 343, and 481 colored according to the SNFG color scheme (46). **(G)** Superposition of backmapped structures from the glycosylated RBD and that in the structure 6VW1. Proteins are colored according to panels **(E)** and **(F)**. RBD Glycosylations sites are shown in magenta, blue, and green for Asn331, Asn343, and Asn481, respectively. Only one conformed every 1 μs is shown. **(H)** Close up on the RBD-ACE2 interaction showing the positions of Asn481 (green), and the closest glycans in ACE2.

Aimed to provide the readers with a brief example of the performance of SIRAH in comparison with fully atomistic simulations, we compared the 15 μs long simulation of SARS-CoV-2 Main protease, present in our database, with an 10 μs long all-atoms simulation of the same protein reported by the Taiji's group and deposited in the Mendeley database (45). Reciprocal (or 2D) RMSD comparison showed that both trajectories visit different conformations with a checkered pattern indicative of conformational fluctuations in both trajectories (Figure 2A). The RMSD using the experimental structure as a reference showed a higher deviation for the CG trajectories of both chains (Figure 2B, bottom), while the gyration radii showed that the CG protein sampled also higher values (Figure 2B, middle). Despite these differences the secondary structure elements were well-maintained during the CG trajectory (Figure 2B, top). The traces for CG and all-atom simulations were similar, although with a loss of nearly 5% in the content of extended beta conformations in the CG case. A superposition against the experimental structure on both trajectories showed roughly comparable features (Figures 2C,D).

A possible use for the dataset is described in the following example. On June 14th, Liu et al. deposited the Cryo-EM structure of the SARS-CoV-2 Spike glycoprotein bound to a human antibody (47). This structure showed that Spike's Receptor Binding Domain (RBD) was glycosylated at Asn331, 343, and 481. Surprisingly, Asn481 neither showed the canonical glycosylation motif nor was previously reported as glycosylated (48). The vicinity of this site to the Angiotensin Converting Enzyme 2 (ACE2) binding zone poses the question of whether glycosylation at Asn481 could modulate the RBD-ACE2 binding (Figures 2E,F). To address this question we took the trajectory of the triple glycosylated RBD from the database (27). First, we used the backmapping capabilities of SIRAH to backmap the trajectory. Second we superimposed the backmapped structures from this CG simulation on the X-ray structure of the RBD-ACE2 complex [PDB id 6VW1 (49)]. This generated an ensemble of possible conformations of glycosylated RBD putatively bound to ACE2, which provided rough insights about possible steric clashes. Despite being close to the ACE2 interface, glycosylation at Asn481 would not be expected to create steric clashes with the human receptor (Figure 2G). Similarly, glycan-glycan interactions at the RBD-ACE2 interface between Asn481 and Asn90, its closest glycosylation site on ACE2 (asterisk in Figures 2E,H) seemed unlikely despite their large flexibility because both moieties remained at opposite sides of the protein-protein interface. Clearly, a thorough analysis would include simulation of complex and all possible glycosylation motifs. Although perfectly possible, this goes beyond the scope of this Data Report.

Nevertheless, besides being useful to foresee large conformational changes and the gross determinants of possible interactions, this kind of information could be useful to decide the expression systems of choice in relation to the length or nature of the glycoforms attainable by prokaryote, insects, or mammalian cells.

Finally, we would like to point out that this is a live initiative and new simulation data will be added. Moreover, we remain open to produce additional simulations upon request.

## Data Availability Statement

The datasets presented in this study can be found in online repositories. The names of the repository/repositories and accession number(s) can be found at: https://zenodo.org.

## Author Contributions

PGG and SP drafted the manuscript. All authors performed MD simulations and contributed to the writing.

## Conflict of Interest

The authors declare that the research was conducted in the absence of any commercial or financial relationships that could be construed as a potential conflict of interest.
